# A silent journey from kidney to lungs: pyelonephritis triggering septic pulmonary embolism – a rare case report from Nepal

**DOI:** 10.1097/MS9.0000000000004307

**Published:** 2025-11-24

**Authors:** Bharat Khatri, Nabin Pahari, Prashant Kunwar, Mukesh Pahari, Shova Aryal

**Affiliations:** a Department of Intensive Care, B.P. Koirala Institute of Health Science, Dharan, Nepal; b Department of Critical Care, Mercy City Hospital, Rupandehi, Nepal; c Department of Pulmonology and Critical Care, AIIMS, New Delhi, India; d Devdaha Medical College, Rupandehi, Nepal; e Department of Emergency, Institute of Medicine, Kathmandu, Nepal

**Keywords:** case report, Klebsiella pneumoniae, pyelonephritis, sepsis, septic pulmonary embolism

## Abstract

**Introduction:**

Septic pulmonary embolism (SPE) is a rare, life-threatening condition caused by infected thrombi from extrapulmonary sites lodging in pulmonary arteries, resulting in infarction and infection. Its nonspecific presentation, including fever, cough, and chest pain, often delays diagnosis. Predisposing factors include infective endocarditis, intravascular catheters, oropharyngeal infections, and urinary tract infections.

**Case presentation:**

A 68-year-old female with hypertension and diabetes mellitus arrived with fever, active cough, sudden shortness of breath, and pain in her left flank. The chest computed tomography (CT) revealed bilateral lung nodules with cavitation, wedge-shaped opacities, and feeding vessel indications, all of which were suggestive of SPE. Laboratory tests revealed leukocytosis and increased C-reactive protein. *Klebsiella pneumoniae* was found in both urine and sputum cultures. The patient was given empirical broad-spectrum antibiotics and ICU care, which included mechanical ventilation. The antibiotic medication was adjusted based on culture sensitivity, resulting in gradual clinical improvement.

**Clinical discussion:**

SPE diagnosis is difficult due to vague symptoms, but can be supported by CT findings such as peripheral nodules, cavitation, wedge-shaped lesions, and feeding vessel indications. Early identification of the major illness source and the use of targeted broad-spectrum antibiotics are crucial. Differential diagnoses include tuberculosis, cancer, necrotizing pneumonia, and fungal diseases; fast cavitation and peripheral spread favor SPE.

**Conclusion:**

SPE should be suspected in patients with extrapulmonary infections presenting with pulmonary nodules or cavitation, particularly when CT shows feeding vessel signs. Prompt diagnosis, removal of infectious sources, and early, appropriate antibiotic therapy are essential to improve patient outcomes.

## Introduction

Septic pulmonary embolism (SPE) is a rare condition that manifests with nonspecific symptoms such as fever, cough, sputum, and pleuritic chest pain, but has a significant mortality^[1]^. Predisposing factors such as infective endocarditis, intravenous drug addiction, venous catheter, tonsillitis, oropharyngeal abscess, and peripheral septic thrombophlebitis (Lemierre’s syndrome) are associated with SPE^[2]^. In the pathogenesis of SPE, inflammation caused by translocation of the microorganism from the primary focus of infection to the venous system, endothelial damage, and thrombogenic toxins produced by the microorganism cause thrombosis. A thrombus leads to infarcts and multiple abscesses where it goes. In patients presenting with fever and nonspecific symptoms and accompanied by a predisposing condition such as the presence of an intravascular catheter, the detection of bilateral nodular and/ or cavitary lesions in thorax computed tomography (CT), which are signs of feeding vessels, nodules or wedge- shaped opacities with basilar and peripheral distribution, should bring SPE to mind, and early antibiotic therapy should be initiated. Treatment principles for SPE include early intravenous broad-spectrum antibiotic therapy and removal of potentially infectious devices (such as intravenous catheters, pacemakers)^[2]^. The mortality rate in SPE is between 10% and 20% and is mainly caused by septic shock and multiorgan failure. Early diagnosis and prompt antibiotic therapy determine the prognosis of the disease^[1–8]^. The exact data for the incidence and prevalence of SPE are not present, but research shows that most of the cases were IV drug users (78%)^[9]^. In a systematic review of 168 cases of SPE, only 7 cases were related to renal infection^[10]^. This shows the rarity and importance of this case. This article has been reported in line with the TITAN 2025 checklist^[11]^.

**HIGHLIGHTS**

- Rare case of pyelonephritis leading to septic pulmonary embolism, underscoring its unusual presentation.
- Septic pulmonary embolism (SPE) can mimic tuberculosis or malignancy; computed tomography findings like feeding vessel signs and peripheral cavitary nodules aid diagnosis.
- Timely recognition and initiation of culture-directed broad-spectrum antibiotics were critical for patient recovery.
- It emphasizes the need to consider SPE in extra pulmonary infections with unexplained pulmonary nodules to improve outcomes.

## Case presentation

**Figure 1.**
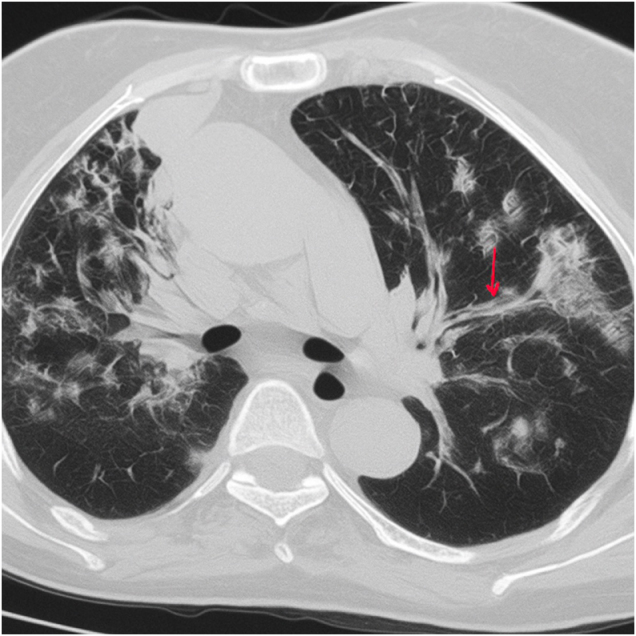
Bilateral multiple lung nodules of variable sizes and some with cavitation and feeding vessels (shown by red arrow) and pleural-based wedge-shaped opacities, which suggested septic pulmonary embolism.

**Figure 2.**
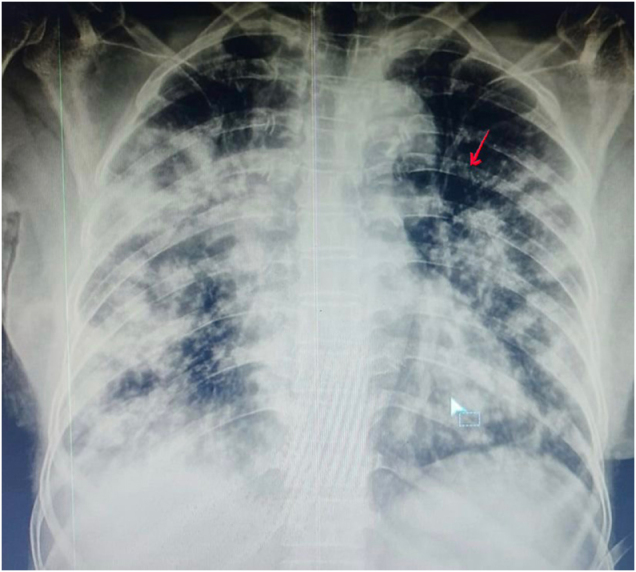
Chest radiograph showing diffuse bilateral nodular densities with cavitation (red arrows).

A 68-year-old female of Rupandehi, Nepal, with a known case of hypertension and diabetes mellitus for 5 years, was referred from a primary healthcare center (PHC) to our tertiary hospital with complaints of fever and productive cough for 2 weeks and sudden onset of shortness of breath and left-sided flank pain for 1 week. She had been recently hospitalized for cough, shortness of breath, fever with chills and rigor, and burning micturition. She was placed on a Foley’s catheter in the PHC. She recalled being extremely weak and frightened when referred to the tertiary hospital. On physical examination, the patient was febrile, tachypneic, and in respiratory distress. On local examination, there was left renal angle tenderness. Chest examination showed active accessory muscles of respiration, stony dull percussion at the middle and base of bilateral lungs, and crackles in bilateral lungs at the infrascapular, infraaxillary, and mammary area. Other systems examination was unremarkable. Chest X-ray revealed bilateral multiple patchy heterogeneous peripheral opacities and infiltrates. Contrast-enhanced computed tomography (CECT) chest showed feeding vessel sign, wedge-shaped opacities, and cavitations, confirming the diagnosis of septic emboli shown in Fig. 1, and chest radiograph showing diffuse bilateral nodular densities with cavitation shown in Fig. 2. Venous Doppler and 2D-echocardiogram were normal. Laboratory findings showed marked leukocytosis 19 000/mm^3^, an acute increase in C-reactive protein (CRP, 13.60 mg/dL), a hemoglobin of 11.8 g/dL, and a platelet count of 163 000/μL were discovered in blood tests, indicating severe inflammation. Arterial blood gas (ABG) showed a pH of 7.29 and a lactate of 4 mmol/L. Urine, blood, and sputum samples were sent for microbiological testing. The patient was admitted to the ICU and was started empirically on piperacillin–tazobactam, azithromycin, linezolid, and nebulization with colistin. On the second day patient got symptomatically improved with decreasing oxygen requirement and work of breathing, but on the third day patient progressively became distressed with tachypnea, tachycardia, and on 14-15 l/min O2 via BiPAP. Her routine investigation got dearranged, which showed AKI and increased leukocytosis 25 000/mm^3^). She was given 500 ml of normal saline 0.9% over 15 minutes intravenous bolus and then was given 30 ml/kg of normal saline (0.9%) over 3 hours. Based on her symptoms, to rule out pulmonary tuberculosis, sputum-based investigations were sent. Sputum Gram stain showed gram-negative rod-shaped bacteria with sterile acid-fast bacilli (AFB) test and negative Gene-Xpert test. Gradually patient became hemodynamically unstable with refractory septic shock and oliguria. The patient was switched to meropenem (dose adjusted according to creatinine clearance). Noradrenaline was started at a dose of 0.1mcg/kg/min and was titrated accordingly. However, by the third ICU day, her condition deteriorated more with acute shortness of breath, tachycardia reaching 120 bpm, and a subsequent decline in GCS, i.e., E2V3M4. The patient was emergency intubated and was given mechanical ventilation support. She needed mechanical ventilation support from the third day to the tenth day of her admission. On the 10th day, most of her blood tests were normal, and she was hemodynamically stable as well with no vasopressors and mechanical ventilation support. Her complete blood count, renal function test, and other blood parameters are shown in a chronological table, i.e., Table 1. Later on, cultures of urine and sputum followed up, which showed *Klebsiella pneumoniae* sensitive to cefoperazone–sulbactam, meropenem, amikacin, and resistant to piperacillin–tazobactam. antibiotics were used on this basis. After extubation, her saturation was maintained to 92% in 5 L/min of oxygen via an oxygen face mask. She was shifted from the ICU to the medical ward on the 15th day of her admission and was discharged home on the 17th day of admission. Upon recovery, she expressed gratitude toward the healthcare team for their continuous care and communication. She emphasized the importance of timely medical evaluation, adherence to diabetic and hypertensive management, and regular follow-up to prevent future complications. She was advised for a 2-week follow-up in which her blood parameters and routine urine test were completely normal. All the chronological events are mentioned in Table 2.

**Table 1:** Blood investigations shown in chronological table

Investigation	1st day of admission	2nd day of admission	10th day of admission	15th day of admission	17th day of admission (discharge day)
Hemoglobin (g/dL)	11.8	11.9	12.4	12.6	12.2
Total leukocyte count (cells/m^3)^	19 000	25 000	15 000	10 800	9900
Neutrophil (%)	86	70	74	81	75
Lymphocyte (%)	8	26	21	14	20
Eosinophil (%)	3	3	3	3	3
Monocyte (%)	3	0	1	2	2
Basophil (%)	0	1	1	0	0
Platelet count (cells/m^3)^	163 000	187 000	256 000	300 000	350 000
Renal function test					
Urea (mg/dL)	30	45	36	32	25
Creatinine (mmol/dL)	1.2	2.5	2	1.1	0.9
Sodium (mmol/L)	133	135	135	138	135
Potassium (mmol/L)	3.8	4	3.5	4.2	4
C-reactive protein (mg/dL)	13.6	14	9	5	2

**Table 2:** Chronological summary of clinical course

Timeline/hospital day	Events and clinical findings	Investigations	Management/treatment	Clinical progress/outcome
Before referral	68-year-old female, known case of hypertension and type 2 diabetes mellitus (5 years). Recently hospitalized for fever, cough, shortness of breath, chills, and burning micturition. Foley’s catheter was placed at the primary healthcare center (PHC).	–	–	Discharged, but symptoms recurred after a few days.
Day 0 (at presentation to tertiary hospital)	Presented with fever and productive cough for 2 weeks and shortness of breath, and left flank pain for 1 week. Physical exam: febrile, tachypneic, respiratory distress, left renal angle tenderness, bilateral crackles, stony dull percussion.	Chest X-ray: bilateral patchy heterogeneous opacities.	Admitted to ICU. Started empirically on piperacillin–tazobactam, azithromycin, linezolid, and colistin nebulization.	Diagnosed as septic embolism with severe sepsis. Stabilized under ICU care.
		CECT chest: wedge-shaped opacities with cavitation and feeding vessel sign suggestive of septic emboli.		
		Venous Doppler and 2D echo: normal.		
		Labs: WBC 19 000/mm^3^, Hb 11.8 g/dL, Platelet 163 000/μL, CRP 13.6 mg/dL, ABG pH 7.29, lactate 4 mmol/L.		
Day 2	Symptomatic improvement with reduced oxygen requirement and respiratory distress.	Routine monitoring.	Continue the same antibiotics and supportive therapy.	Slight improvement noted.
Day 3 (early)	Worsening symptoms – tachypnea, tachycardia, respiratory distress, requiring 14–15 L/min O₂ via BiPAP.	WBC: 25 000/mm^3^; Acute kidney injury detected.	Antibiotic changed to meropenem (dose adjusted).	Refractory septic shock with oliguria developed.
			Noradrenaline infusion started (0.1 µg/kg/min, titrated).	
		Sputum Gram stain: gram-negative rods.		
		AFB and GeneXpert: negative.		
Day 3 (later)	Condition further deteriorated with tachycardia (120 bpm), acute SOB, and GCS drop (E2V3M4).	–	Emergency intubation and mechanical ventilation were initiated.	Shifted to full ventilatory and inotropic support.
Days 3–10	ICU stay with gradual improvement in hemodynamics and respiratory parameters.	Serial blood tests and renal function are monitored.	Continued meropenem, fluids, vasopressors, and supportive care.	Gradual recovery noted.
Day 10	Improved clinically; no distress.	CBC, RFT, and CRP normalized.	Off mechanical ventilation and vasopressors.	Extubated successfully, stable on oxygen support.
Days 10–15	Steady recovery in ICU.	Urine and sputum culture: *Klebsiella pneumoniae* sensitive to cefoperazone–sulbactam, meropenem, amikacin; resistant to piperacillin–tazobactam.	Antibiotics are modified based on culture sensitivity.	Maintained SpO₂ 92% on 5 L/min oxygen via face mask.
Day 15	Clinically stable and afebrile.	–	Shifted from the ICU to the medical ward.	Continued to improve.
Day 17 (discharge)	Stable vitals and normal laboratory parameters.	CBC, RFT normal.	Discharged on maintenance therapy.	Advised follow-up after 2 weeks.
2-week follow-up	Asymptomatic and doing well.	Blood parameters and urine routine are normal.	–	Complete recovery.

## Discussion

SPE is a rare lung illness that is characterized by infection, infarction, and bilateral nodules and/or cavities in the lung parenchyma as a result of a microorganism-containing thrombus embolising into the venous circulation and settling in the pulmonary arteries. Intravenous drug use, infective endocarditis, intravascular catheters, hemodialysis, pacemaker, periodontal abscess, and liver abscess are among the reasons that cause SPE^[3]^. Its clinical presentation may range from nonspecific symptoms such as fever, cough, sputum, and pleuritic chest pain to acute sepsis^[4]^. Therefore, it is difficult to diagnose, and hence, the diagnosis of the disease may be delayed. The diagnosis is mostly made with the presence of clinical findings associated with the infection, the presence of predisposing factors, and CT findings. Thorax CT is superior to chest X-ray in both diagnosis and differential diagnosis^[5]^. Typical CT findings may include nodules and/or cavities located mostly in the area close to peripheral vessel terminations (a sign of feeding vessel; Fig. 1), infiltrates, wedge-shaped lesions adjacent to the pleura, pleural effusion, focal consolidation, and abscess^[6–8]^. The most critical factor for disease prognosis is to consider SPE and start broad-spectrum antibiotic therapy early^[3–5]^. This case, who was referred with an initial diagnosis of tuberculosis due to cavitary lung lesions and whose empirical antibiotic therapy was delayed at the time of referral, was presented together with the literature to highlight SPE in the differential diagnosis of multiple cavities and to draw attention to the removal of the catheter, which is thought to be the source of infection, and to the initiation of early antibiotic therapy. SPE should be considered when the primary focus of infection is found to be accompanied by high fever and nodules and/or cavities located in the area close to the multiple peripheral vessel endings in the lungs. While the finding of a feeding vessel indicates that the lesion is of hematogenous origin, the presence of cavitation is an indicator of infarction^[5]^. Although these findings support the SPE diagnosis, they can also be seen in other lung diseases. However, the feeding vessel sign is very sensitive for SPE. The differential diagnosis of cavitary nodules includes lung cancer, tuberculosis, hydatid cysts, fungal infections, necrotizing pneumonia, Wegener’s granulomatosis, and rheumatological diseases such as rheumatoid arthritis. Rapid cavitation of nodules in SPE helps differentiate them from malignancy. Cavitary lesions dominant on the peripheral lower lobe, randomly distributed, and with a feeding vessel sign, in particular, support the SPE diagnosis as in our case, while cavitary lesions in the upper lobe apical region, consolidation, tree-in-bud pattern, and galaxy sign can be considered tuberculosis^[7]^. Treatment principles for SPE include early intravenous broad-spectrum antibiotic therapy and removal of potential sources of infection (such as intravenous catheters). In SPE, mortality is between 10 and 20% and is mainly caused by septic shock and multiorgan failure^[3–6]^. SPE can also indirectly cause acute kidney injury through systemic sepsis, hypotension, and inflammation, or directly through embolic involvement of renal vasculature^[12]^. In conclusion, SPE is an uncommon disease with a high fatality rate. When a patient presents with fever and nonspecific symptoms and has a predisposing condition, such as the presence of an intravascular catheter, the identification of bilateral nodules and/or cavitations with a feeding vessel sign on thorax CT should alert SPE. The disease’s prognosis is determined by early diagnosis and timely treatment.

## Conclusion

This example demonstrates how a condition of pyelonephritis can gradually progress to a life-threatening complication such as SPE. The patient’s trip demonstrates that generic symptoms, such as fever and cough, can mask serious underlying illnesses. Careful attention to imaging characteristics, such as peripheral cavitary nodules and feeding vessel indications on CT, along with rapid, focused antibiotic therapy, can mean the difference between survival and death. Early venous imaging (CT/MR venography) helps detect renal vein thrombosis before embolization. Prompt antibiotic therapy and abscess drainage are critical for reducing bacterial burden and thrombus development. When thrombosis is verified, antibiotic-based anticoagulation may prevent septic embolism. Multidisciplinary care and close respiratory monitoring aid in the early detection and treatment of complications such as SPE. We hope that by publishing this instance, we might enhance clinician knowledge and encourage vigilance and fast response in similar situations, thereby saving lives.

## Data Availability

The dissemination of the article data is freely accessible.
